# Author response to: Letter to Editor in Response to the association between contact with children and the clinical course of COVID-19

**DOI:** 10.1017/S0950268822001133

**Published:** 2022-07-11

**Authors:** Peter Jannuzzi, Gregory A. Panza

**Affiliations:** 1 Integrated Care Partners, Hartford HealthCare, Hartford, CT, USA; 2 Unionville Pediatrics, LLC, Unionville, CT, USA; 3 Department of Research, Hartford HealthCare, Hartford, CT, USA

We thank Soyemi and Soyemi for their thoughts and insights regarding our paper. They begin their letter accurately summarising the overall finding of our study. They also note that hospitalisation was lower for patients with multiple concurrent child contact types (home and workplace) compared with patients with no contact (20 (12%) *vs.* 72 (43.1%), respectively), and find this surprising because of the likelihood of infection from multiple sources. This observation is correct; however, it is important to understand that hospitalisation by contact type was not statistically compared within the hospitalised group so it is difficult to make any inferences for such a comparison, especially without considering any covariates. In addition, the likelihood of infection (all participants in our study were SARS-CoV-2 positive) or where the infection came from was not examined in our study because this information was unnecessary to examine the study research question. Rather than transmission of the *novel* virus from child to adult, one of the theories from which our study purpose and hypothesis derived was that children may have had prior minor coronavirus infections leading to immunity or resistance to the novel virus [1–3], and perhaps this resistance may be present in adults who have had more contact with children prior to getting infected. Therefore, we were interested in where the children contact occurred and the magnitude of contact rather than if the transmission of SARS-CoV-2 occurred from child to adult as a result of the contact, although it is possible that this occurred in some cases. Based on this theory, if we did aim to examine where and how transmission occurred and if infections were occurring from child to adult via the workplace and home, we would have still hypothesised that the adults who were infected as a result of contact with children at multiple sources would have had a lower rate of hospitalisation than SARS-CoV-2-positive patients with no contact due to their pre-infection child contact from these multiple sources.

Similar to Soyemi *et al*., we were surprised by the main finding of our study, as it did not support our hypothesis when we adjusted for covariates and was therefore inconsistent with previous studies examining this research question. We do believe this is likely due to the methodological limitations of previous studies (e.g. lack of controlling for all appropriate covariates, descriptive analysis only, non-verified self-reported data) [4–6] which we describe in the introduction of our paper. We aimed to improve upon these limitations by (1) collecting more extensive demographic and health data, (2) objectively verifying these data with patient charts and (3) controlling for all appropriate potential covariates that may influence the clinical course of COVID-19. We mention in the discussion that in non-randomised study designs it is essential to control for potential confounders in an attempt to avoid type I error and the importance of this may be evident by our results that showed significance or trends that support a protective effect for the ‘home contact’ primary outcome, but was no longer significant after adjusting for covariates.

We agree with Soyemi *et al*. that it is important to understand the role of children in the spread of SARS-CoV-2. However, we would like to re-emphasise that examining the spread of SARS-CoV-2 from child to adult or vice versa was not an aim of our study. Soyemi *et al*. reference a study (Ustundag *et al*. [7]) in their letter that displayed different findings than our study. Ustundag *et al*. examined if contact with a household family member with SARS-CoV-2 was associated with clinical outcomes (e.g. hospitalisation) in patients aged 1 month to 18 years with SARS-CoV-2. We feel that the purpose of Ustundag *et al*.'s study was considerably different than ours, with a different population being studied, and a different association being examined. As a result, it is very difficult to comment on why we did not find higher hospitalisation in adults with SARS-CoV-2 that had contact with children (infection status not tracked), while Ustundag *et al*. found higher hospitalisation in children with SARS-CoV-2, who had contact with a household family member with SARS-CoV-2.

Soyemi *et al*. makes several great points regarding the importance of understanding the transmission of SARS-CoV-2 and we agree that there is heterogeneity in the literature regarding the infectivity of children. Soyemi *et al*. references two studies [8, 9] in their letter that indicate transmission rates are similar in children and adults. Contrary to these two studies, a recent study by Bullard *et al*. [10] found that children who tested positive for SARS-CoV-2 via nasopharyngeal swab were less likely to grow virus in culture, had higher cycle thresholds and lower viral concentrations compared to adults with SARS-CoV-2. The authors conclude that these findings suggest children are not the primary drivers of SARS-CoV-2 transmission. However, it does not appear that the number of exposures children had was accounted for in this study and as Soyemi *et al*. stated, greater contact/exposure frequency may balance lower infectivity. Another recent study by Bhatt *et al*. [11] examined household transmission among symptomatic and asymptomatic children and adults exposed to SARS-CoV-2 in their households and found that children were responsible for one-third of the household spread. Based on these continued inconsistencies in the literature, we agree that additional research is needed to better understand the transmission dynamics of children which remains critical in the prevention of spread in households, schools and daycares.
